# Nutritive Value, In Vitro Fermentation, and Methane Production of Cactus Cladodes, Sugarcane Bagasse, and Urea

**DOI:** 10.3390/ani11051266

**Published:** 2021-04-28

**Authors:** Michelle Siqueira, Juana Chagas, João Paulo Monnerat, Carolina Monteiro, Robert Mora-Luna, José Dubeux, Nicolas DiLorenzo, Martin Ruiz-Moreno, Marcelo Ferreira

**Affiliations:** 1 Department of Animal Science, Federal Rural University of Pernambuco, Dom Manoel de Medeiros Street, Dois Irmãos, Recife, Pernambuco 52171-900, Brazil; Michelle.siqueira2@gmail.com (M.S.); joao.monnerat@ufrpe.br (J.P.M.); robertmora78@yahoo.com (R.M.-L.); marcelo.aferreira@ufrpe.br (M.F.); 2 Department of Agricultural Research for Northern Sweden, Swedish University of Agricultural Sciences (SLU), 901 83 Umeå, Sweden; juana.chagas@slu.se; 3 Animal Science Department, State University of Alagoas, BR 316, km 87,5, Bebedouro, Santana do Ipanema 57500-000, Brazil; 4 National Experimental University of Táchira, Sector 5001, San Cristóbal, Venezuela; 5 Department of Animal Science, North Florida Research and Education Center, University of Florida, 3925 Hwy 71, Marianna, FL 32446-8091, USA; dubeux@ufl.edu (J.D.J.); ndilorenzo@ufl.edu (N.D.); ruizm001@ufl.edu (M.R.-M.)

**Keywords:** *Cactaceae*, energy, methane, semiarid, small ruminant, waste

## Abstract

**Simple Summary:**

Cyclical droughts affecting arid and semiarid regions promote direct negative impacts on agriculture, with deficits of water availability for the maintenance of crops destined for human supply and animal production, with direct and indirect socioeconomic effects. Although livestock rearing is one of the few viable economic activities for these regions, forage production in terms of quantity and quality is a critical obstacle to support the herd over the year. Research was developed to find a forage adapted to these climates. Cactus cladodes have been used as a traditional ingredient in ruminant diets in dry areas as a solution to forage scarcity. Many traditionally used sources of forage, fresh or conserved, can be added to diets with cactus. However, the limiting factor to their inclusion would be market availability and price. This study showed that cactus cladodes associated with sugarcane bagasse (a high available crop residue) present the same nutritive value of conserved crops as silages and hay. Cactus (*Opuntia* and *Nopalea*) are essential for ruminant production systems in arid and semiarid regions due to the limitation of forage production caused by the low precipitation.

**Abstract:**

This study evaluated the effect of different roughages fed to sheep on nutrient and water intake, ingestive behavior, nitrogen balance, microbial protein synthesis, fermentation parameters, and methane production using an in vitro gas production system. The treatments consisted of five diets: cactus cladodes *Nopalea* (NUB) and *Opuntia* (OUB), both with the addition of sugarcane bagasse (SB) and urea/ammonium sulfate (urea/as); Tifton hay (TH); corn silage (CS); and sorghum silage (SS), also with added urea/as. The NUB provided greater (*p* ≤ 0.03) intakes of dry matter (1024 g/d), digestible organic matter (670 g/d), and crude protein (161 g/d) than those on the SS. The NUB provided greater (*p* < 0.01) dietary water intake (3023 g/d) than TH. The time spent on rumination was shorter (*p* < 0.01), and the idle time was longer in animals fed NUB and OUB than TH and CS. Microbial protein synthesis was not affected (*p* = 0.27). The final pH (6.4) of the incubation fluid and the concentration of NH_3_-N (39.05 mg/dL) were greater for NUB and OUB. Ruminal parameters and methane production were little or not affected by tested forages. We recommend using cactus cladodes in combination with sugarcane bagasse and urea/as in sheep diets.

## 1. Introduction

Meteorological and hydrological droughts are known phenomena that affect arid and semiarid regions. The first occurs when dry weather patterns dominate an area, and the second occurs when low water supply becomes evident, especially in reservoirs and groundwater levels. Both promote direct negative impacts on agriculture, with deficits of water availability for the maintenance of crops destined for human supply and animal production, with direct and indirect socioeconomic effects [1,2]. Although livestock rearing is one of the few viable economic activities for these regions, forage production in terms of quantity and quality is a critical obstacle to support the herd over the year. Cactus cladodes have been adopted globally in dry areas as a basis for ruminant diets [3], especially regarding great energy content (2.38 Mcal of ME/kg of DM) [4], and high water content, which minimizes the effect of water scarcity. The high intake of cactus cladodes (proportion in diet around 500 g/kg of DM) contributes to meet the water requirement of dairy cows, sheep, and goats [5]. A combination of cactus cladodes with sources of nitrogen (e.g., urea) and effective fiber is recommended to guarantee ideal rumen conditions and macronutrient supplies to meet the nutritional requirements of ruminants [6].

Many traditionally used sources of forage, fresh or conserved, can be added to diets with cactus; however, the limiting factor to their inclusion would be market availability and price. In attempts to solve this problem, many types of roughage are used, such as sugarcane, silages, grass hay, legumes (Brazil), *Atriplex halimus* L. (Syria), *Prosopis laevigata* ssp. (Mexico), leguminous hay (Zimbabwe), and wheat straw (Tunisia) [3,7,8,9]. Despite the excellent results verified by combining these feeds with cactus cladodes, their use greatly increases production system costs due to high production and transport costs. As a result of the high energy concentration of cactus, it is possible to adopt ingredients presenting low nutritional value and commercialized at lower prices. In this sense, sugarcane bagasse, an agricultural waste composed of 80% neutral detergent fiber, appears as a feasible option.

The hypothesis was that cactus cladodes associated with sugarcane bagasse and urea would show similar nutritional value to commonly conserved roughage adopted in the semiarid region. The aims of this research were (1) to evaluate the effect of different types of roughage fed to sheep on nutrient and water intake, ingestive behavior, nitrogen balance, and ruminal microbial protein synthesis; further, (2) to evaluate the effect of roughage on fermentable parameters and methane production using an in vitro gas production system.

## 2. Materials and Methods

The study was conducted in accordance with the standards of the National Council for Control of Animal Experimentation (CONCEA) and approved by the Ethics Committee on Use of Animal for Research (CEUA; License No. 069/2016).

### 2.1. Experiment 1: In Vivo

This study was carried out in the Department of Animal Science at the Federal Rural University of Pernambuco, located in Recife, Pernambuco, Brazil.

### 2.2. Animals and Diets

Five rumen fistulated sheep with average initial body weight (BW) of 34.0 ± 3.63 kg, housed in individual pens (0.93 × 1.54 m²) fitted with feeders and waterers were assigned in a 5 × 5 Latin square design, with five consecutive 22-day periods divided into 14-day adaptation and 8-day sampling periods.

The diets were offered *ad libitum* as total mixed ration, allowing orts about 100 g/kg of dry matter (DM) offered. The animals were fed twice a day at 0800 and 1600 h. The experimental diets were formulated in a roughage:concentrate ratio of 69.4:30.6. The experimental diets consisted of five roughages: cactus *Nopalea cochenillifera* (L.) Salm-Dyck cladodes (*Nopalea*) + urea/as + sugarcane bagasse (NUB), cactus *Opuntia stricta* (Haw.) Haw cladodes (*Opuntia*) + urea/as + sugarcane bagasse (OUB), Tifton hay (TH; *Cynodon* spp), and corn silage (CS; *Zea mays* L.—Agroceres^®^ AG5055 cultivar: yellow-orange and medium grain of short cycle) and sorghum silage (SS; *Sorghum bicolor* L. Moench—SF-15 variety). The crude protein (CP) concentration of TH (92.0 g/kg) was adopted as a standard for all diets, so the other diets had the percentage of CP corrected with urea/sa (9:1; Table 1 and Table 2).

### 2.3. Data and Sample Collection

Feed and orts were weighed daily throughout the experimental period for the calculation of nutrient intake. The concentration of organic matter (OM) was calculated by the difference between DM and ash concentrations.

Voluntary water intake (VWI) was calculated by the difference between the offered and the leftover water, carried out over three days, totaling 72 h, corrected by the evaporation rate, following the formula: Voluntary water intake = (leftover water) − evaporated water. The water evaporation rate was measured using four buckets positioned in the center of the shed.

Observations about the ingestive behavior were performed using the instantaneous scanning method proposed by [10]. The animals were observed every 10 min, starting immediately after morning feeding, totaling 24 h of observation in each period. The activities were recorded for each animal, feeding (FT), rumination (RT), idle (IT), and chewing activities (CT; feeding time + rumination time). Feeding and ruminating efficiencies of DM and NDF (g/min) were calculated as in [11]. Feed bunk efficiency (g DM/feed bunk visit) = the amount of dry matter intake for each feed bunk visit.

Fecal collections were performed from the 16th to the 18th day of each experimental period to estimate total apparent dry matter digestibility and its nutrients, using collecting bags attached to the body of the animals.

During this period (16th to 18th day), total urine collection was performed through 24 h, measuring the urine pH every six hours to maintain it below 3.0. The collectors were attached to the foreskin of the animal to conduct urine to a bottle containing 100 mL of 10% sulfuric acid. At the end of each collection period, the weight and total volume of urine were determined [12]. A 50-mL aliquot was frozen at −20 °C for chemical analysis.

The nitrogen balance (NB) was obtained through the difference between nitrogen intake (Ni), and nitrogen excreted in feces (Nf) and urine (Nu).

On the 18th day of each experimental period, four hours after morning feeding were taken blood samples from the jugular vein with 21Gx1 needles (Vacuette^®^, Greiner Bio-One, Kremsmünster, Austria), using Vacuette^®^ tubes (Greiner Bio-One, São Paulo, Brazil) with anticoagulant (heparin) for further analysis of plasma urea nitrogen concentration (PUN).

The purine derivatives (PD) excreted were calculated as the sum of daily urinary excretion of allantoin and uric acid, without considering xanthine and hypoxanthine excretion, because allantoin + uric acid is highly correlated with rumen nucleic acid concentration [13]. The PD absorbed was calculated according to the mathematical model described by [14]:

Y = 0.84X + 0.150BW^0.75^ e^−0.25X^ (1)

where: Y is the excretion of purine derivatives (mmol/day); X corresponds to the absorbed microbial purines (mmol/day); BW^0.75^ corresponds to the body weight raised to the 0.75 power (metabolic weight). The microbial N supply (MNS) was estimated, according to [15]:

MNS (g/d) = X (mmol/day) × 70/0.116 × 0.83 × 1000 = 0.727X(2)

Assuming a digestibility of 0.83 for microbial purines, a ratio of 0.116 for purine N:total N, and a N content of purines of 70 mg N/mmol. The microbial protein supply (MPS) was calculated (MNS × 6.25).

### 2.4. Chemical Analysis and Composition

At collection days, roughage, concentrate, and orts were sampled and stored in plastic bags at −20 °C. At the end of the experiment, the samples were oven-dried at 60 °C for 72 h and ground to pass through a 2-mm mesh for in situ ruminal incubation and through a 1-mm screen for further chemical analyses.

Dry matter (method 934.01), ash (method 942.05), crude protein (method 968.06), and ether extract (EE; method 920.39) were analyzed according to [16]. Subsequently, NDF was analyzed using heat-stable α-amylase (Termomyl^®^, 2X) as described by [17], and acid detergent fiber (ADF) as described by [18]. Neutral detergent insoluble nitrogen (NDIN) was analyzed using the Kjeldahl method [19]. The iNDF content was analyzed using the fecal, feeds, and orts samples processed at 2-mm screen sieve, obtained by using in situ procedures with 288 h of rumen incubation in cattle, as described by [20].

Non-fiber carbohydrates (NFC) were calculated according to [21]:

NFC = 100% − [%ash + %EE + %NDF+ (%CP − %CPu + %U)](3)

where CPu = CP content from urea, U = urea content. For microbial synthesis efficiency. (%). The digestibility of dry matter and nutrients were calculated using the equation:

Nutrient digestibility = (nutrient intake − nutrient in feces)/nutrient intake(4)

The total digestible nutrients (TDN) were estimated using the equation:

TDN = dCP + dEE × 2.25 + dNFC + dNDF(5)

where, d = digestible; and metabolizable energy (ME) was estimated as described by [22].

The concentration of urea, creatinine, and uric acid were determined using commercial kits (LABTEST^®^). The microbial protein synthesis was estimated by determining purine derivatives concentration in urine by the colorimetric method, as described by [12].

### 2.5. Experiment 2: In Vitro Incubation and Gas Production

All in vitro study procedures were conducted at the North Florida Research and Education Center (NFREC; Marianna, FL, USA).

### 2.6. Feed Sample Collection and Analyses

The proportion of ingredients and chemical composition of the samples are described in Table 2. These combinations of treatments were used to evaluate in vitro DM (IVDMD) and OM digestibility (IVOMD), gas and methane (CH_4_) production, and ammonia nitrogen (NH_3_-N), and volatile fatty acid (VFA) concentrations. Each ingredient was air-dried for at least 72 h and ground to pass through a 2 mm screen (urea was ground using a mortar and pestle) in a Wiley mill (Arthur H. Thomas Co., Philadelphia, PA, USA).

### 2.7. In Vitro Incubations and Analysis

The samples were incubated for 48 h at 39 °C under constant agitation (60 rpm) in 125-mL bottles, and gas production was recorded using the Bottle System (NFREC, Marianna, FL, USA). The readings were measured at 0, 12, 24, and 48 h after incubation. Pressure data (psi = pounds per square inch) were converted to the volume of gas (V) by the equation:

V = 5.1612P − 0.3017, R^2^ = 0.9873 (6)

Generated in the Production Laboratory (LPG) of the Federal University of Agreste of Pernambuco, UFAPE, from 937 observations. Bottles containing 0.7 g of the samples and 50 mL of inoculum were incubated for 48 h, and VFA concentrations were measured at the end of the incubation period along with NH_3_-N concentrations, pH, and CH_4_ production. Incubations were repeated on three periods, and two bottles per treatment were incubated in each period. At the end of the incubation period, the fermentation was stopped by adding 0.5 mL of 20% H_2_SO_4_ solution to each bottle. Immediately afterwards, a 10 mL sample was taken and frozen for subsequent VFA and NH_3_-N analyses. Ruminal fluid used for inoculum was collected from two ruminally cannulated Angus crossbred steers (601 ± 16.0 kg BW). These animals received a diet based on Tifton hay and concentrate (corn and soybean meal) with a roughage:concentrate ratio of 70:30, for at least 2 weeks before the collection of ruminal fluid. Ruminal fluid was strained from a representative sample of digesta through four layers of cheesecloth, placed in a prewarmed thermos container, and transported to the laboratory within 30 min of collection. A 3:1 McDougall’s buffer:ruminal fluid mixture was used as inoculum for incubations.

### 2.8. Methane Measurements

Gas samples were taken from each bottle at 0, 12, 24, and 48 h of incubation using a 60 mL syringe and were later transferred to another previously evacuated container (−25 mm Hg). CH_4_ concentrations were analyzed by gas chromatography (Agilent 7820A GC; Agilent Technologies, Palo Alto, CA, USA) using flame ionization and a capillary column (Plot Fused Silica 25 m × 0.32 mm, Coating Molsieve 5A, Varian CP7536; Varian Inc., Lake Forest, CA, USA). Certified standards were used to standardize the gas chromatograph for CH_4_ (4 mg/L, Scott Marrin Inc., Riverside, CA, USA). For CH_4_ analyses, injector, column, and detector temperatures were 80, 160, and 200 °C, respectively, and N_2_ was used as the carrier gas with a flow rate of 3.3 mL/min. The split ratio for the injected CH_4_ sample was 5:1.

Cumulative CH_4_ production at each time point was calculated according to the equation [23]:

TM = HSVol × HSmet + GP × A × HSmet(7)

where TM = total CH_4_ (mL); HSVol = headspace volume (mL); HSmet = headspace CH_4_ concentration; GP = gas production (mL); and A is the ratio (0.55) of CH_4_ concentration in outflow gas to headspace. The total headspace in the system was 125 mL. The total gas volume was automatically recorded by the system and corrected for the normal air pressure.

### 2.9. Ammonia Nitrogen, pH, and Volatile Fatty Acids

At 48 h of incubation, the bottles were removed from the water bath, and pH was measured immediately. After pH measurement, a composite sample of each replicate of fluid was mixed with H_2_SO_4_ (20%) and stored at 20 °C for analysis of NH_3_-N and VFA. Concentrations of NH_3_-N in the incubation fluid were measured after centrifuging at 10,000× *g* for 15 min at 4 °C (Avanti J-E; Beckman Coulter Inc.) following the phenol-hypochlorite technique described by [24] with the following modification: absorbance was read at 620 nm in flat-bottomed 96-well plates using a plate reader (DU-500; Beckman Coulter Inc.). VFA in the samples were determined in a water-based solution using an ethyl acetate extraction. Samples were centrifuged for 10 min at 10,000× *g* at 5 °C. Five milliliters of the ruminal fluid supernatant was mixed with 0.5 mL of a meta-phosphoric acid:crotonic acid (internal standard) solution, and samples were frozen overnight, thawed, and centrifuged for 10 min at 10,000× *g* at 5 °C. The supernatant was transferred into vials and mixed with ethyl acetate in a 2:1 ratio of ethyl acetate to the supernatant. After vigorously shaking vials, the ethyl acetate fraction rose to the top, and a subsample was transferred to a vial. Samples were analyzed by gas chromatography (Agilent 7820A GC; Agilent Technologies, Palo Alto, CA, USA) using a flame ionization detector and a capillary column (CP-WAX 58 FFAP 25 m × 0.53 mm, Varian CP7767; Agilent Technologies, Inc., Santa Clara, CA, USA).

### 2.10. In Vitro Dry Matter and Organic Matter Digestibility

IVDMD was measured using 100 mL plastic scintillation bottles with rubber stoppers fitted with a 16-gauge needle for gas release. Tared bottles containing 0.7 g of the substrate and 50 mL of a 3:1 solution of McDougall’s buffer:ruminal fluid for substrates were incubated for 48 h at 39 °C under constant agitation (60 rpm). Two bottles per treatment and two blank bottles (without substrate) were incubated in each of three separate replicate periods. Afterwards, the residue was filtered to obtain a solid fraction. After this procedure, the solid part was placed in a forced-air ventilation oven at 105 °C for 24 h and weighed to calculate the remaining DM. To calculate the IVDMD of each bottle, the dry residue weight (corrected for the contribution of solids in the blank) was subtracted from the incubated DM, and the result (i.e., digested DM) was divided by the dry weight of substrate incubated. After this process, the residue was placed in a muffle furnace at 550 °C for 6 h to obtain IVOMD.

### 2.11. Statistical Analysis

The data were analyzed using the MIXED procedure of the SAS software (SAS Institute Inc., Cary, NC, version 9.2), according to the following model:

Yijk = m + Ti + aj + Pk + eijk(8)

where, Yijk the observation in animal j, submitted to treatment i, during period k; μ = the overall mean; Ti = effect of treatment i (fixed effect); aj = animal effect j (random effect); Pk = effect of the period k (random effect); and eijk is the unobservable random error.

The IVDMD, IVOMD, gas production, methane, NH_3_–N, pH, and VFA data were analyzed using the MIXED procedure of SAS, with a block (incubation period) as a random effect, with incubation sample (the average of two replicas per day of each incubated sample) as the experimental unit

All averages were compared using the Tukey test, with a critical level of 5% of probability for type I error.

## 3. Results

### 3.1. Experiment 1

#### 3.1.1. Intake of Nutrients

The NUB diet provided greater intake of DM (1024 g/d), digestible OM (DOM; 670 g/d), and CP (161 g/d) than the SS diet (*p* < 0.03; Table 3). NDF intake (*p* < 0.01) was lesser for animals submitted to OUB treatment (310 g/d) when compared with TH and CS (525 and 422 g/d, respectively). The animals consuming the NUB and OUB diets showed the greatest (*p* < 0.01; Table 3) water intake via diet (3023 and 2439g/d, respectively) and consequently drank less water from the water trough (996 and 1158 g/d, respectively). The total volume of water intake was similar across all treatments (*p* = 0.07).

#### 3.1.2. Ingestive Behavior

The feeding time and feeding and ruminating efficiencies of NDF (NDF intake/FT or RT), CT, and feed bunk efficiency did not vary among roughages (*p* ≥ 0.08; Table 4). The ruminating time (466 and 436 min/d) was shorter (*p* < 0.01), and the idle time was longer (*p* < 0.01; 542 and 578 min/d) for NUB and OUB, respectively, when compared with TH and CS. OUB and SS treatments presented the least values for number of meals (*p* < 0.01) related to TH. The NUB diet provided greater (*p* ≤ 0.04) feeding and ruminating efficiency of DM (299; 132 g DM/h, respectively) compared with SS (188 and 91 g DM/h, respectively).

#### 3.1.3. Nitrogen Balance and Microbial Protein Synthesis

Animals fed with NUB showed a greater N intake (25.7 g/d), and lower values of N excretion via feces (*p* ≤ 0.01; Table 5) were for treatments composed by NUB, OUB, and SS (4.4, 4.2, and 4.3 g/d, respectively) compared with TH and CS (5.6 and 6.5, respectively). Consequently, animals fed with NUB showed greater NB (*p* = 0.01) than those fed with SS. The greatest value for plasma urea N was for animals fed with NUB, and the least was for those fed with CS (*p* > 0.01). Microbial synthesis and efficiency were not different (*p* = 0.27) among the evaluated roughages.

### 3.2. Experiment 2

#### 3.2.1. In Vitro Digestibility and VFA Proportion

Corn silage presented the greatest IVDMD and IVOMD (*p* < 0.01), and TH presented the least (Table 6). Total VFA production did not differ between treatments. NUB and OUB presented the greatest molar proportion of butyrate, and the greatest A/P ratio and (A + B)/P (*p* < 0.01) were greater for NUB, OUB, and TH. Tifton hay presented the greatest molar proportion of acetate (*p* < 0.01) while CS presented the least. Corn silage and SS provided the greatest propionate concentration (*p* < 0.01). Moreover, CS presented the greatest values for isobutyric, valeric, and isovaleric acids (*p* < 0.01), and SS presented the least values for all VFA proportions, except for acetate and propionate (Table 6).

#### 3.2.2. In Vitro Gas Production and pH

Ammonium concentration and pH (*p* < 0.01) were greater for NUB and OUB than the other diet. The total production and concentration of CH4 were similar for all diets. Finally, gas production at 0, 12, 24, and 48 h (*p* < 0.01) was greater for diets with cactus (NUB and OUB; Table 6).

## 4. Discussion

### 4.1. Experiment 1

The greater DM intake observed for NUB compared with SS can be attributed to the great concentration of NFC present in cactus cladodes [25] and to feed processing. According to [26], cactus cladodes processed in a forage machine can stimulate intake because they expose mucilage. Therefore, the other poorly palatable feed components, such as urea and sugarcane bagasse, which can adhere to the cactus cladodes, avoid selection by animals. OM, CP, and DOM intake presented a similar response to DM intake (Table 1).

Another important observation was the low quality of the SS, highlighting the reduced levels of DM and NFC (230 g/kg on an as-fed basis and 164 g/kg of DM) and the great levels of NDF and ADF (676 and 469 g/kg DM; Table 1). The great concentration of ADF may compromise DM digestibility, providing the effect of rumen repletion and decreased intake [27].

The voluntary water intake was lesser due to the great moisture concentration of both cactus cladodes (881 g/kg on average) compared with TH (162 g/kg). Other studies with cactus cladodes reported decreased voluntary water intake by animals [28,29]. These reports demonstrated that cactus such as *Opuntia* or *Nopalea* are highly important to the water supply in semiarid regions, where water has low quality and limited quantity.

Despite the difference in DM intake, the time spent on feeding was similar for all diets, which probably indicates a selection attempt by the animals due to the low quality of the SS diet. The lower NDF concentration and intake of treatments with NUB and OUB justified the shorter ruminating time and longer idle time presented. However, the use of diets with a great proportion of fiber will imply more time for chewing and, particularly, for performing rumination activity, as was verified in this study for TH and CS (Table 2, Table 3 and Table 4).

The greater feeding and rumination efficiency (g DM/h) observed for NUB vs. SS (Table 5), despite the similar feeding times, was mainly due to the greater intake of DM in less time for the treatment with NUB (Table 3). [30] found an increase in feed efficiency up to 364 g/kg DM of cactus cladode inclusion as a replacement for roughage with a greater NDF concentration (TH). Ruminating activity is related to feed quality, intake amount, and fibrous material amount in the rumen. Thus, ruminating efficiency could be explained by the lesser NDF concentration in cactus cladode treatments.

In tropical and other warm regions, the nutritional value of livestock feed must be considered, mainly regarding the energy content. The processes of rumination and digestion are related to the calorie increment and the reduction in the efficiency of metabolizable energy use to maintain the animals [22]. Thus, the addition of cactus cladodes in ruminant diets can reduce heat production by decreasing rumination and increasing caloric increment [31]. Moreover, the energy spent on thermoregulatory processes should be redirected to the production of meat or milk.

The greater nitrogen intake verified for NUB (25.7 g of N/day) compared with SS was due to the greater DM intake, so the PUN concentration for all diets corresponded to nitrogen intake. According to [32], the degradation of non-protein nitrogen (NPN) in the rumen is <300%/h. This greater amount of NPN with NUB resulted in a greater concentration of ammonia in the rumen and, consequently, provided an increase in PUN level (Table 5) [33].

The greatest PUN concentration (24.3 mg/dL; Table 5), verified 4 h after the feed supply, is within the range (24.0 to 50.0 mg/dL) proposed as ideal for sheep [34], thus reflecting in better use of N by animals fed with the NUB diet. The concentration of ruminal NH3–N and the PUN content are related to ammonia absorption into the portal blood [35].

The different roughages did not influence microbial protein synthesis and efficiency (Table 5). Although roughages vary in their proportions of fibrous and non-fibrous carbohydrates, as well as their rates of degradation, they had a greater concentration of degradable protein in the rumen (urea and soybean meal). In this way, a synchronism between the availability of protein and energy for microbial protein synthesis can be proposed.

### 4.2. Experiment 2

The best IVDMD and IVOMD being provided by CS, NUB, and OUB may be explained mainly due to their great NFC concentration. These carbohydrates are fermented rapidly by microorganisms that improve the digestibility of the diets. An additional factor is the reduced iNDF concentration in those diets. However, one of the NUB and OUB ingredients is sugarcane bagasse, which has high iNDF. The great NFC content of spineless cactus compensates for the low digestibility of sugarcane bagasse, and it allows NUB and OUB to present similar DM and OM in vitro digestibility compared with corn silage (Table 6).

The similarity in VFA concentrations is reflected in the similar potential of all diets. The values of potential VFA reported for NUB and OUB in this study show the potential of cactus cladodes with sugarcane and urea as an alternative to traditional roughage used in the semiarid region. The highest acetate concentration for TH is a result of greater NDF and lower NFC content. The lowest pH for CS and SS resulted from propionate concentration that significantly increased, which led to a tendency increase in total VFA concentration, and a significant decrease in the ratio of A/P, which changed the pattern of rumen fermentation [36]. NH_3_-N was higher for NUB and OUB because 20 and 19 g/kg of urea were added, respectively. Ruminal pH is an indication of the balance between the level of ammonia and total VFA in the rumen. The similar total VFA concentrations and greater ruminal ammonia concentration with urea supplementation explain the greater pH for NUB and OUB (Table 6). Urea is rapidly fermented to ammonium by microorganisms in the rumen; as these diets received more urea [37], they consequently obtained a greater ammonia concentration.

The similar CH_4_ production for all diets probably is due to the same roughage:concentrate ratio (69.4:30.6). The roughage:concentrate ratio is one of many factors that affect CH_4_ production. According to [38], great inclusion of grain in the ruminant diet lowers enteric CH_4_ production. Another relevant fact is that the NH_3_-N production (27.6 mL/g of DM) for all diets was below than those published by [39] when using a control diet with timothy grass (545 g/kg of DM), rolled barley (363 g/kg of DM), and rapeseed (92 g/kg of DM); they obtained methane production of 39.7 mL/g of DM.

The diets composed of cactus cladodes, sugarcane bagasse, and urea presented the greatest gas production from 12 h; this probably resulted from more NH_3_-N production from these treatments (Table 6).

All diets were formulated to be balanced. So, according to [40], forage quality, feeding a balanced diet to ensure efficient utilization of nutrients, and optimized microbial protein synthesis in the rumen can decrease CH_4_ production in relation to animal productivity. The methane data revealed in this paper are the first demonstration of the potential of using this source of roughage in semiarid regions. Brazil is one of the most important countries for livestock production, and responsibility for the environment is one criterion for production systems. Mitigation of methane production by livestock ruminants in the semiarid region is now being initiated.

## 5. Conclusions

Both cactus cladodes associated with sugarcane bagasse and urea showed a similar TH and CS nutritional value, mostly regarding energy intake and nitrogen efficiency, and they were superior to SS. Another important point is that sugarcane bagasse and urea are easy to buy ingredients and they are marketed at low prices. The animals fed with cactus cladodes with sugarcane bagasse and urea needed less water supply than those fed with TH and SS. Also, they spent less time ruminating compared with others tested conserved forages. Additionally, ruminal parameters and methane production were minimally or not affected by tested forages. Therefore, we recommend using cactus cladodes in combination with sugarcane bagasse and urea as an alternative to conserved forages in sheep diets.

## Figures and Tables

**Table 1:** Chemical composition of ingredients used in the experimental diets (g/kg DM).

Iten	*Nopalea*	*Opuntia*	Sugarcane Bagasse	Tifton Hay	Corn Silage	Sorghum Silage	Ground Corn	Soybean Meal
Dry matter	116	122	911	838	249	230	879	888
Organic matter	876	887	954	914	940	917	983	929
Crude Protein	34.0	40.0	11.0	92.0	89.0	60.0	76.0	497
Indigestible crude protein	9.2	9.3	8.5	39.5	12.7	13.1	1.22	138
Neutral detergent fiber	260	302	823	728	590	676	142	141
Indigestible neutral detergent fiber	97.0	119	456	296	181	229	16.0	14.0
Non-fiber carbohydrates	568	532	114	89.0	245	164	724	279

**Table 2:** Proportion of ingredients and chemical composition of the experimental diets.

Item	Diet				
	NUB ^1^	OUB ^2^	TH ^3^	CS ^4^	SS ^5^
Ingredients (g/kg DM)					
Cactus *Nopalea*	379	--	--	--	--
Cactus *Opuntia*	--	375	--	--	--
Tifton hay	--	--	694	--	--
Corn silage	--	--	--	692	--
Sorghum silage	--	--	--	--	683
Ground corn	175	175	175	175	175
Soybean meal	115	115	115	115	115
Sugarcane bagasse	295	300	--	--	--
Urea/as ^6^	20	19	00	02	11
Mineral mix	16	16	16	16	16
Diet composition (g/kg of DM)					
Dry matter ^7^	253	267	853	323	300
Organic matter	893	898	913	929	906
Crude protein	140	139	134	137	141
Ether extract	15.3	15.7	12.3	18.9	20.2
Neutral detergent fiber	382	401	546	449	503
Indigestible neutral detergent fiber	176	185	209	129	161
Non-fiber carbohydrates	386	375	220	374	283
Total digestible nutrients	712	664	582	641	626
Metabolizable energy (Mcal/kg DM)	25.7	24.0	21.0	23.2	22.6

^1^ NUB = *Nopalea* + urea + sugarcane bagasse; ^2^ OUB = *Opuntia* + urea + sugarcane bagasse; ^3^ TH = Tifton hay; ^4^ CS = Corn silage; ^5^ SS = Sorghum silage; ^6^ Urea + ammonium sulfate (9:1); ^7^ g/kg as fed.

**Table 3:** Nutrient and water intake for sheep fed different roughages.

Item	Diet						
	NUB ^1^	OUB ^2^	TH ^3^	CS ^4^	SS ^5^	SEM	*p*-Value
Intake (g/day)							
Dry matter	1024 a	888 ab	993 ab	982 ab	781 b	51.1	0.03
Organic matter	903 a	792 ab	906 a	913 a	705 b	46.3	0.03
Crude protein	160 a	136 ab	140 ab	142 ab	117 b	19.8	<0.01
Neutral detergent fiber	334 bc	310 c	525 a	422 ab	381 bc	24.3	<0.01
Non-fiber carbohydrates	433 a	360 ab	208 c	326 b	205 c	18.5	<0.01
Digestible organic matter	669 a	550 ab	594 ab	614 ab	469 b	39.7	0.03
Water intake (g/day)							
From diet	3023 a	2439 ab	171 c	2060 b	1824 b	208	<0.01
Voluntary water	996 b	1158 b	3129 a	1926 b	1877 b	202	<0.01
Total	4019	3597	3301	3986	3702	151	0.07

^1^ NUB = *Nopalea* + urea + sugarcane bagasse; ^2^ OUB = *Opuntia* + urea + sugarcane bagasse; ^3^ TH = Tifton hay; ^4^ CS = Corn silage; ^5^ SS = Sorghum silage. BW: body weight. Means followed by different letters on the same line differ by the Tukey test (*p* < 0.05).

**Table 4:** Ingestive behavior for sheep fed different roughages.

Item	Diet						
	NUB ^1^	OUB ^2^	TH ^3^	CS ^4^	SS ^5^	SEM	*p*-Value
Feeding time (min/day)	224	202	302	264	256	23.5	0.08
Rumination time (min/day)	466 b	436 b	596 a	598 a	520 ab	25.5	<0.01
Idle time (min/day)	750 a	802 a	542 b	578 b	664 ab	35.5	<0.01
Feed bunk visit	8.2 ab	7.4 b	10.2 a	9.6 ab	7.4 b	16.8	<0.01
Feed bunk efficiency (g DM/feed bunk event)	131	124	108	102	113	33.4	0.35
Total chewing time							
(min/day)	690 b	638 b	898 a	862 a	776 ab	35.5	<0.01
(g DM/hour)	90.0 a	84.3 ab	67.3 abc	71.0 bc	60.9 c	44.8	<0.01
(g NDF/hour)	29.3	29.4	35.6	30.5	29.7	19.8	0.19
Efficiencies, (g/h)							
Feeding efficiency (DM) ^6^	299 a	268 ab	209 ab	248 ab	188 ab	24.4	0.04
Feeding efficiency (NDF) ^6^	97.9	93.6	110	106	91.3	29.3	0.55
Rumination efficiency (DM) ^6^	132 a	124 ab	101 ab	102 ab	90.8 b	23.8	0.01
Rumination efficiency (NDF) ^6^	42.8	43.2	53.6	44.0	44.2	33.6	0.19

^1^ Cactus *Nopalea* + urea + sugarcane bagasse; ^2^ Cactus *Opuntia* + urea + sugarcane bagasse; ^3^ Tifton hay; ^4^ Corn silage; ^5^ Sorghum silage. Means followed by different letters on the same line differ by the Tukey test (*p* < 0.05); ^6^ Dividing the intake of each of these nutrients by the total feeding time (feed efficiency) and rumination time (rumination efficiency).

**Table 5:** Nitrogen balance (N), urea nitrogen concentration, and microbial protein synthesis for sheep fed different roughages.

Item	Diet					SEM	*p*-Value
	NUB ^1^	OUB ^2^	TH ^3^	CS ^4^	SS ^5^		
N balance, g/day							
N intake (g/d)	25.7a	21.8ab	22.4ab	22.7ab	18.7b	0.99	<0.01
N faecal (g/d)	4.4b	4.2b	5.6a	6.5a	4.3b	0.23	<0.01
N urinary (g/d)	8.8	8.7	8.1	8.6	9.1	1.18	0.98
N balance (NB)	12.5a	8.9ab	8.7ab	7.6ab	5.3b	1.14	0.01
NB (% of intake)	48.6	40.8	38.8	33.4	28.4	4.36	0.06
Plasma Urea Nitrogen, mg/dL	24.3a	21.2b	20.6b	17.1c	19.6bc	0.67	<0.01
Microbial Protein Synthesis							
Microbial N (g/d)	6.3	5.0	4.8	5.6	4.9	46.3	0.40
Microbial efficiency (g MN ^6^/kg TDN)	51.58	60.18	52.56	56.32	57.90	28.0	0.27

^1^ Cactus *Nopalea* + urea + sugarcane bagasse; ^2^ Cactus *Opuntia* + urea + sugarcane bagasse; ^3^ Tifton hay; ^4^ Corn silage; ^5^ Sorghum silage; ^6^ Microbial nitrogen Means followed by different letters on the same line differ by the Tukey test (*p* < 0.05).

**Table 6:** Effect of experimental diets on in vitro fermentation and methane production.

Item	Diet					SEM	*p*-Value
	NUB ^1^	OUB ^2^	TH ^3^	CS ^4^	SS ^5^		
IVDMD, g/kg	662 ab	672 ab	617 c	680 a	636 bc	9.2	<0.01
IVOMD, g/kg	649 abc	664 ab	609 c	671 a	627 bc	11.4	<0.01
VFA, mM/L	93.7	91.7	90.9	95.7	95.4	4.2	0.08
Acetate, mM/mol	65.2 b	65.1 b	66.1 a	63.4 d	64.3 c	0.56	<0.01
Propionate, mM/mol	20.2 b	20.0 b	19.9 b	21.9 a	22.1 a	0.47	<0.01
Butyrate, mM/mol	10.4 a	10.7 a	9.7 bc	10.3 ab	9.6 c	0.925	<0.01
Isobutyrate, mM/mol	1.0 bc	1.0 abc	1.0 ab	1.0 a	0.9 c	0.014	<0.01
Valerate, mM/mol	1.3 ab	1.3 ab	1.4 a	1.4 a	1.3 b	0.045	0.02
Isovalerate, mM/mol	1.9 ab	1.9 ab	1.9 ab	2.0 a	1.8 b	0.088	0.02
A/P	3.2 a	3.2 a	3.3 a	2.9 b	2.9 b	0.051	<0.01
(A + B)/P	3.7 a	3.8 a	3.8 a	3.4 b	3.4 b	0.104	<0.01
pH	6.41 a	6.40 a	6.34 b	6.27 c	6.32 bc	0.059	<0.01
NH_3_-N (mg/dL)	39.3 a	38.8 a	26.2 b	23.9 b	25.5 b	2.5	<0.01
CH_4_							
48 h, mL/g DM	23.6	24.0	23.5	23.9	22.2	2.6	0.80
mL/L of Gas	176.3	178.8	175.3	178.1	165.7	19.5	0.82
Gas production (ml/g DM) as a function of collection times							
0 h	4.80 a	4.53 ab	3.10 b	3.8 ab	3.8 ab	0.568	0.027
12 h	36.1 a	35.0 a	26.2 b	33.1 ab	30.0 b	1.7	<0.01
24 h	85.1 a	83.1 a	68.1 c	82.4 ab	74.6 bc	4.1	<0.01
48 h	142.2 a	140.0 a	125.0 b	142.7 a	132.2 ab	6.9	<0.01

^1^ Cactus *Nopalea* + urea + sugarcane bagasse; ^2^ Cactus *Opuntia* + urea + sugarcane bagasse; ^3^ Tifton hay; ^4^ Corn silage; ^5^ Sorghum silage. Means followed by different letters on the same line differ by the Tukey test (*p* < 0.05).
